# Effect of epidural blood injection on upright posture intolerance in patients with headaches due to intracranial hypotension: A prospective study

**DOI:** 10.1002/brb3.1026

**Published:** 2018-06-19

**Authors:** Adnan I. Qureshi, Danish Kherani, Muhammad A. Waqas, Mushtaq H. Qureshi, Faisal M. Raja, Shawn S. Wallery

**Affiliations:** ^1^ Zeenat Qureshi Stroke Institute St. Cloud Minnesota; ^2^ University of Illinois and Mercyhealth Rockford Illinois

**Keywords:** cerebrospinal fluid leakage, epidural blood injection, headaches, intracranial hypotension syndrome, upright posture intolerance, visual analogue scale

## Abstract

**Background:**

We performed a prospective study to quantify changes in various aspects of upright posture intolerance in patients with intracranial hypotension.

**Methods:**

Six patients were provided a standard questionnaire before, immediately after epidural blood patch injection and at follow‐up visit within 1 month after epidural blood injection inquiring: (a) How long can they stand straight without any support? (b) Do they feel any sense of sickness when they sit or lie down after standing? (c) How long do they have to wait before they are comfortable standing again after they have stood straight? (d) How effectively and fast can they get up from sitting or lying position to stand straight? and (e) Rate their activities in upright posture without support on a standard vertical visual analogue scale between 100 (can do everything) and 0 (cannot do anything).

**Results:**

All patients responded that they could not stand straight for ≥30 min (four responding <5 min) on pretreatment evaluation. All patients reported improvement in this measure immediately postprocedure with two reporting ≥30 min. At follow‐up, three patients reported further improvement and one patient reported worsening in this measure. The magnitude of improvement ranged from 10 to 80 points increase immediately postprocedure in their ability to perform activities, while they are standing without any support on visual analogue scale. At follow‐up, four patient reported additional improvement in their ability to perform activities, while they are standing without any support (ranged from 10 to 20 points increase compared with immediately postprocedure rating).

**Conclusions:**

We present semiquantitative data on various aspects of upright posture intolerance in patients with intracranial hypotension before and after epidural blood injection.

## 1 INTRODUCTION

Orthostatic headaches are one of the features of intracranial hypotension syndrome (Schievink, Maya, Louy, Moser, & Tourje, 2008; Schievink et al., 2011) which are characterized by headache that significantly worsens soon after sitting upright or standing and/or improves after lying horizontally. The symptoms are secondary to low cerebrospinal fluid (CSF) pressure (Headache Classification Committee of the International Headache Society (IHS), 2013) occurring immediately after assuming an upright position and resolving quickly after lying horizontally. There is a delayed variant where worsening in symptoms occurs after minutes or hours of being upright, but not necessarily resolving, after minutes or hours of lying horizontally (Juraschek et al., 2017; Singer & Low, 2017; Sunwoo et al., 2017). Due to orthostatic headaches, orthostatic tolerance or tolerance to upright posture is expected to be impaired among patients with intracranial hypotension syndrome. Orthostatic intolerance is a phenomenon described in patients with postural hypotension (Abidi et al., 2017), postural tachycardia (Heyer, 2017; Thanavaro & Thanavaro, 2011), cerebellar and spinal cord‐related ataxias (Claydon & Krassioukov, 2006; Miwa & Inoue, 2017; Schwabova et al., 2012), and old age‐related delay in activation of muscle contraction (Oddsson, 1990). Orthostatic intolerance has been associated with an increased risk of fall, fracture, syncope, and motor vehicle accidents (Finucane et al., 2017).

We performed this prospective study to characterize the orthostatic or upright posture intolerance in patients with headaches due to intracranial hypotension and identify changes following epidural blood injection.

## 2 METHODS

All consecutive patients treated for intracranial hypotension by interventional neurology team at Mercy health Rockford hospital between 1 July and 14 November 2017 were evaluated. The patients met the criteria for postlumbar puncture headaches subcategory of headache attributed to low CSF pressure as defined in *International Classification of Headache Disorders, 3rd edition (beta version)* (Headache Classification Committee of the International Headache Society (IHS), 2013). The patients were provided with a standard questionnaire to inquire regarding various aspects of upright posture intolerance as previously described (Qureshi, 2018). The questionnaire inquired regarding the following questions: (a) How long can they stand straight without any support; (b) Do they feel any sense of sickness (headache, nausea, dizziness, lightheadedness etc.) when they sit or lie down after standing straight with or without support; (c) How long do they have to wait before they are comfortable standing again after they have stood straight with or without support? There were four response categories: (a) Not at all (or cannot stand straight without any support); (b) <5 min; (c) 5–29 min; and (d) 30 min or greater. The questionnaires consist of structured, forced, multiple‐choice responses (Grimmer & Williams, 2000; Kuorinka et al., 1987; Lipton et al., 2007). Another question inquired regarding how effectively and fast can they get up from sitting or lying position to stand straight without any support. There were three response categories: (a) Can stand up easily without interruption or support; (b) Have to stand up from sitting or lying position with interruption (slow) or in two steps or more, and (c) Must hold on to temporary support (<5 min). The patients were asked to rate their ability to perform activities such as household chores (housework), office work, writing, reading, eating, toilet activities while they are standing without any support. The respondents were asked to rate their activities on a standard vertical visual analogue scale (similar to a thermometer) between 100 (can do everything, best imaginable) and 0 (cannot do anything worst imaginable). The visual analogue scale methodology is based on a psychometric response scale which has been used in questionnaires to ascertain subjective characteristics that cannot be directly measured. The data are inputted based on the rank ordering of scores rather than their exact values (Gift & Narsavage, 1998; Wewers & Lowe, 1990). The scale was provided in 10‐point increments as previous studies have suggested a change of 10 points or greater as clinically significant (Mishra et al., 2015; Spertus et al., 1995). The analysis was descriptive and presented as individual patient data (Table 1).

**Table 1:** Changes in various aspects of upright posture intolerance before, immediately after, and at 1‐month follow‐up in response to epidural blood injection

	How long can you stand straight without any support?	Do you feel any sense of sickness (headache, nausea, dizziness, lightheadedness, etc.) when you sit or lie down after standing straight with or without support?	How long do you have to wait before you are comfortable standing again after you have stood straight with or without support?	How effectively and fast can you get up from sitting or lying position to stand straight without any support?	Ability to perform activities such as household chores, office work, writing, reading, eating, toilet activities while standing without support (0‐ cannot do anything [worst] to 100‐ Can do everything [best])
Patient 1					
Preprocedure	<5 min	5–29 min	<5 min	Must hold on to temporary support (<5 min) to stand up from sitting or lying position	40
Postprocedure	5–29 min	5–29 min	5–29 min	Have to stand up from sitting or lying position with interruption (slow) or in two steps or more	50
1‐month follow‐up	5–29 min	Not at all	5–29 min	Have to stand up from sitting or lying position with interruption (slow) or in two steps or more	70
Patient 2					
Preprocedure	5–29 min	30 min or greater	30 min or greater	Have to stand up from sitting or lying position with interruption (slow) or in two steps or more	50
Postprocedure	30 min or greater	Not at all	Not at all	Have to stand up from sitting or lying position with interruption (slow) or in two steps or more	80
1‐month follow‐up	30 min or greater	Not at all	Not at all	Can stand easily without interruption or support	100 [best]
Patient 3					
Preprocedure	<5 min	<5 min	Not at all	Must hold on to temporary support (<5 min) to stand up from sitting or lying position	0 [worst]
Postprocedure	5–29 min	5–29 min	5–29 min	Have to stand up from sitting or lying position with interruption (slow) or in two steps or more	50
1‐month follow‐up	30 min or greater	Not at all	30 min or greater	Must hold on to temporary support (<5 min) to stand up from sitting or lying position	50
Patient 4					
Preprocedure	Cannot stand straight without the support (below the knee amputation)	<5 min	<5 min	Must hold on to temporary support (<5 min) to stand up from sitting or lying position	0 [worst]
Postprocedure	<5 min	<5 min	<5 min	Can stand up easily without interruption or support	40
1‐month follow‐up	5–29 min	30 min or greater	<5 min	Can stand up easily without interruption or support	50
Patient 5					
Preprocedure	<5 min	30 min or greater	<5 min	Have to stand up from sitting or lying position with interruption (slow) or in two steps or more	0 [worst]
Postprocedure	5–29 min	Not at all	5–29 min	Can stand up easily without interruption or support	80
1‐month follow‐up	30 min or greater	Not at all	Not at all	Can stand up easily without interruption or support	100 [best]
Patient 6					
Preprocedure	<5 min	<5 min	<5 min	Must hold on to temporary support (<5 min) to stand up from sitting or lying position	10 (with headache) otherwise 90 (without headache)
Postprocedure	30 min or greater	Not at all	Not at all	Can stand up easily without interruption or support	70 (with headache) otherwise 80 (without headache)
1‐month follow‐up^a^	5–10 min	30 min or greater	5–29 min	Have to stand up from sitting or lying position with interruption (slow) or in two steps or more	40

a Follow‐up was performed earlier at 10 days postprocedure.

## 3 RESULTS

### 3.1 Patient 1

A 61‐year‐old woman presented with a persistent headache for 6 days which started after she received an epidural steroid injection for chronic back pain. The patient had no history of any similar headaches in the past. The headache was described as severe and throbbing. The headache was worse with activity and upright position and improved in the supine position. She rated the severity as 8/10 at peak intensity. The patient underwent magnetic resonance imaging of the brain with gadolinium enhancement that showed the mild descent of cerebellar tonsils through foramen magnum but no dural enhancement. Magnetic resonance venogram was unremarkable showing no evidence of venous sinus thrombosis. The patient underwent epidural blood injection and reported improvement in severity of headaches during upright posture to 5/10 in severity. Headache frequency and intensity were decreased over next 2 days after which she was discharged on pain medication.

### 3.2 Patient 2

A 25‐year‐old woman underwent fluoroscopic‐guided lumbar puncture for new‐onset occipital headaches primarily on the right side occasionally radiating to the temporal region. The initial pressure was 10 mmHg by fluid‐coupled measurements and 11 cm of H_2_O by fluid column method. A total of 18 cc of clear CSF was obtained. The final pressure was 6 mmHg by fluid‐coupled measurements and 7 cm of H_2_O by fluid column method. The patient developed headaches (rated as 10/10 in severity) that were exacerbated by upright posture postprocedure. The headache continued for 5 days postprocedure and had not improved with hydromorphone, acetaminophen, decadron, indomethacin, lorazepam, caffeine sodium benzoate, and hydrocodone‐acetaminophen treatment. The patient underwent an occipital nerve block which did not reduce the severity of the positional headaches. Magnetic resonance imaging with gadolinium enhancement demonstrated protrusion of cerebellar tonsils through the foramen magnum, slit ventricles, and enhancement of dura. The patient underwent epidural injection of whole blood and reported complete resolution of headaches (0/10) and was able to ambulate without any difficulty.

### 3.3 Patient 3

A 20‐year‐old woman had a progressively worsening headache since delivery and epidural anesthesia at lumbar 4 and 5 vertebral level 5 days ago. The headache was initially frontal but became diffuse and was associated with nausea and vomiting. The headache was described as 9/10 in severity and worse on standing position and interfered with activities of daily living and sleep. The patient received ibuprofen, intravenous caffeine, caffeine tablets, ketorolac without any significant relief. Magnetic resonance imaging with gadolinium enhancement demonstrates cerebellar tonsils descending through the foramen magnum, slit ventricles, and enhancement of dura. The patient underwent epidural injection of whole blood and reported complete resolution of headaches (0/10) immediately postprocedure.

### 3.4 Patient 4

A 34‐year‐old woman with past medical history of complex regional pain syndrome developed a frontal headache which progressively worsened over a period of 7 days. She developed fever and nausea and underwent a lumbar puncture as part of the diagnostic evaluation. Multiple attempts were made to sitting position for performing a lumbar puncture and CSF pressure was not measured. She reported worsening headache with localization in the high cervical segment with extension into the occipital and frontal regions after lumbar puncture accompanied by photophobia, nausea, and chest pain. Headache was worse in an upright position with the rated severity of 10/10 and reduced to 5/10 in severity in lying position. Magnetic resonance imaging with gadolinium enhancement demonstrates cerebellar tonsils descending through the foramen magnum, slit ventricles, and enhancement of dura. The patient received oxycodone‐acetaminophen, intravenous caffeine, tramadol, and butalbital‐acetaminophen‐caffeine without any relief. The patient underwent epidural injection of whole blood and reported an immediate complete reduction in of headache severity (4/10) in an upright position with a resolution of photophobia.

### 3.5 Patient 5

A 28‐year‐old man underwent fluoroscopic‐guided lumbar puncture as part of the evaluation of new‐onset diffuse headaches for several weeks ago which developed after coitus. The initial pressure was 5 mmHg by fluid‐coupled measurements and 11.5 cm of H_2_O by fluid column method. A total of 10 cc of clear CSF was removed. The final pressure was 2 mmHg by fluid‐coupled measurements and 3.5 cm of H_2_O by fluid column method. After lumbar puncture, he developed a headache which was different from an original headache with clear worsening when the patient was upright. The severity was rated as 8/10 and accompanied by nausea and vomiting. The headache was persistent for 4 days and had not improved with steroids, caffeine infusion, and hydrocodone‐acetaminophen treatment. Magnetic resonance imaging with gadolinium enhancement demonstrates cerebellar tonsils descending through the foramen magnum, slit ventricles, and enhancement of dura. The patient underwent epidural injection of whole blood and reported complete resolution of headaches (0/10) and was able to ambulate without any difficulty.

### 3.6 Patient 6

A 34‐year‐old woman presented with severe episodic headaches that started after giving birth to a child 5 months ago which was rated 5/10 in severity. The headaches were associated with nausea, vomiting, and photophobia with no clear relationship to posture. The patient also reported visual scotomas in both the eyes. Magnetic resonance imaging demonstrated findings of empty sella syndrome but there was no distortion of optic nerves or posterior aspect of the optic globe. The patient had a fluoroscopic‐guided lumbar puncture performed to measure CSF pressure to exclude intracranial hypertension. The opening CSF pressure was 7 mmHg by fluid‐coupled measurements and 15 cm of H_2_0 by fluid column method. A total of 16 cc of clear CSF was removed. The final pressure was 3 mmHg by fluid‐coupled measurements and 5 cm of H_2_O by fluid column method. The patient reported worsening of headaches postprocedure. The patient described it as a continuous and excruciating headache that progressively worsened was rated as 10/10 in severity. A noncontrast magnetic resonance imaging demonstrated cerebellar tonsils descending into the foramen magnum. A contrast‐enhanced computed tomographic scan did not demonstrate any dural enhancement. The patient received caffeine tablets, topiramate, morphine injection, intravenous hydromorphone, and caffeine infusion during admission for pain but had no significant relief. The patient underwent epidural injection of whole blood and reported complete resolution of headaches (0/10) and photophobia after the procedure and was able to ambulate without any difficulty.

## 4 PROCEDURE

Every patient underwent epidural blood patch using left paramedian lumbar approach under fluoroscopic guidance in the prone position. An 18 gauge (3.5 or 6 inches) Reganes spinal needle was inserted between spinous processes and laminae of L3 and L4 or L4 and L5 vertebrae (one level below the previous lumbar puncture when relevant) under fluoroscopic guidance. A 3 cc syringe was attached to the spinal needle with gentle pressure. The loss of resistance method with the 3 cc syringe was used to localize the epidural space while advancing the spinal needle. Approximately, 3 cc of radio‐opaque contrast (ISOVUE‐M‐200, Iopamidol) was injected and the epidural space was identified with linear cephalad opacification of posterior epidural space. A total of 25 cc of autologous venous blood (with 5 cc of Iopamidol) was injected into epidural space.

## 5 ASSESSMENT OF UPRIGHT POSTURE TOLERANCE

All patients responded that they could not stand straight for 30 min or greater with four responding that they can stand straight for <5 min. Patient 4 reported partial inability due to below knee amputation. All patients reported improvement in this measure immediately postprocedure with two reporting ability to stand straight without support for 30 min or greater. At follow‐up, three patients reported further improvement and one patient reported worsening in this measure.

All patients reported that they have a sense of sickness (headache, nausea, dizziness, lightheadedness) when they sit or lie down after standing straight with or without support with two reporting that the sense of sickness persisted for 30 min or greater. Three patients reported improvement, two no change, and one worsening immediately after the procedure. Four patients at follow‐up reported that they did not have any sense of sickness when they sit or lie down after standing straight.

Five of 6 patients reported that they have to wait before they are comfortable standing again after they have stood straight with or without support with four reporting <5 min. Interesting, three patients reported worsening and two patients reported improvement in this measure before they are comfortable standing again immediately after the procedure. Only two patients at follow‐up reported that they do not have to wait before they are comfortable standing again after they have stood straight with or without support.

All patients reported difficulty in the query regarding how effectively and fast can they get up from sitting or lying position to standing straight without any support with four reporting that must hold on to temporary support (<5 min) to stand up from sitting or lying position and two reporting that they have to stand up from sitting or lying position with interruption (slow) or in two steps or more. Three patients reported no difficulty and two reported improvements in the ability to query regarding effectively and fast can they get up from sitting or lying position to stand straight without any support immediately after treatment. Two patients reported deterioration and three patients reported no difficulty to the query regarding how effectively and fast can they get up from sitting or lying position to stand straight at follow‐up.

All patients rated their ability to perform activities such as household chores (housework), office work, writing, reading, eating, toilet activities while they are standing without any support as <100 (can do everything). Three rated the query as 0 (worst, cannot do anything) and other three rated the query as 10, 40, and 50. All patients reported improvement in their ability to perform activities while they are standing without any support immediately postprocedure. The magnitude of improvement ranged from 10‐ to 80‐point increase immediately postprocedure. At follow‐up, four patient reported additional improvement in their ability to perform activities while they are standing without any support. The magnitude of improvement ranged from 10‐ to 20‐point increase compared with immediately postprocedure rating on visual analogue scale. One patient reported no further change and one patient reported 30 points worsening compared with the immediately postprocedure rating. All patients rated an improvement in their ability to perform activities at follow‐up compared with pretreatment evaluation ranging from 30 to 100 points on visual analogue scale.

## 6 DISCUSSION

The current study presents data on various aspects of upright posture intolerance in patients with headaches due to intracranial hypotension before and after epidural blood injection using a semiquantitative assessment. Previous studies have reported the effectiveness of epidural blood injection on orthostatic headaches and hearing difficulties in patients with intracranial hypotension (Ferrante, Olgiati, Sangalli, & Rubino, 2016; So et al., 2016; Wu et al., 2017). Our study provides additional information regarding another important aspect of intracranial hypotension syndrome, which is upright posture intolerance. We assessed the duration of ability to stand and ability to perform activities when standing straight without support. We also assessed the pace of standing up from supine or sitting position. Two additional items evaluated the hangover from standing straight without support. The magnitude of improvement varied among patients and some components demonstrated more consistent change. All patients reported impairment in ability to stand straight for 30 min or greater, a sense of sickness when they sit or lie down after standing straight, and suboptimal performance in query regarding how effectively and fast can they get up from sitting or lying position to standing straight before treatment. Five of 6 patients reported that they have to wait before they are comfortable standing again after they have stood straight with or without support before treatment. All patients rated their ability to perform activities while they are standing without any support as <100 (can do everything) visual analogue scale. However, the treatment response was consistently seen as an increase in the time duration of ability to stand straight without support and improvement in the rating of ability to perform activities while they are standing on visual analogue scale. The improvement in these items was seen on immediate postprocedure assessment and additional improvement was seen on follow‐up assessment. The treatment response was most inconsistent with additional items that evaluated the hangover from standing straight without support which may be attributed to psychological components determining performance in such measures.

The study has certain limitations which need to be considered prior to interpretation. The study design was a case series method where measurements are within‐individuals and rely on self‐matched controlling that eliminate all fixed confounders. Case series are helpful in understanding the unique aspects of an individual patient or disease and may lead to new concepts in clinical medicine (Albrecht, Meves, & Bigby, 2005; Vandenbroucke, 2001). The number of patients is small due to the relatively infrequent occurrence of intracranial hypotension syndrome. Previous studies have reported among 15–20 patients per year in large hospitals (Karm et al., 2016; Schievink et al., 2008; Wu et al., 2017). The assessment of upright posture intolerance was performed prospectively to avoid any recall bias where patients may remember their former state as better or as worse than it actually was and thus confound the magnitude of improvement (Blome & Augustin, 2015; Karm et al., 2016; McPhail & Haines, 2010). The influence of current state (depending upon how good the patient feels at the time of assessment) can sometimes cause the patient to amplify the magnitude of change by rating the preprocedure state lower retrospectively compared with if the rating was done prospectively. There was no control population, and the magnitude of benefit with medical treatment alone is not quantified. However, epidural injection of blood is considered an integral part of the management of intracranial hypotension syndrome (Karm et al., 2016; Schievink et al., 2008; Wu et al., 2017), and withholding treatment may not be considered appropriate.

We provide new data regarding various aspects of upright posture intolerance in patients with headaches due to intracranial hypotension and response to epidural blood injection. We hope that such measures will be incorporated into future studies involving patients with intracranial hypotension syndrome and other disease processes with upright posture intolerance.

## DISCLOSURES

The authors do not have any conflict of interests to disclose.
